# Extracts from Cit. Lombart's Observations on Chirurgical Cases, &c.

**Published:** 1799-09

**Authors:** 


### *Extracts from Cit.* LOMBART'*s Obſervations on Chirurgical Caſes, &c.*

[Continued from our laſt Number, pp. 46—50.

AMONG theſe obſervations (ſays the French editor), the ſixth and ſeventh are particularly worthy of attention, as they evince the danger of attempting to cure particular ſpecies of *fiſtula ani*, eſpecially when Nature appears to have made choice of this canal to diſcharge matter, the retention of which would neceſſarily effect the diſſolution of the patient.

### SIXTH OBSERVATION.

A Clergyman of Réthel-Mazarin was afflicted for a number of years with a ſuffocative aſthma, in which he had a remiſſion of four hours only in the four and twenty, and this after an abundant expectoration. The ſymptoms of the diſeaſe left not the ſmalleſt doubt of the ſtate of the patient. In the ſpring of 1783 he made a journey on horſeback. This exerciſe occaſioned an abceſs at the extremity of the anus, which was ſucceeded by a fiſtula. Four months after this happened I was called in (ſays Cit. LOMBART), and as I knew his ſtate previous to the appearance of the fiſtulous ulcer, I perſuaded him to conſider this ſymptom as a ſalutary effort of Nature to relieve him from a diſeaſe which would ſoon have terminated his life. I remarked to him that he was no longer troubled with his habitual aſthmatic affection; that the expectoration which had undermined his conſtitution had now abated; that his reſpiration was free, his lungs ſound; and that he had again acquired muſcular fulneſs and energy; that his ſleep was no longer interrupted; and in ſhort, that if he wiſhed to continue in a good ſtate of health, he muſt relinquiſh the idea of curing his fiſtula. Soon afterwards the patient changed his reſidence, and came to Meſiny. I was informed that he had reſolved to undergo the operation. I forewarned him of the danger to which he would expoſe himſelf; and adviſed him to nominate an executor, if he was determined to venture on the operation. However, no credit was given to my ſuggeſtions, nay the officiating ſurgeon treated them as merely chimerical. At length the clergyman underwent the operation, but beneficent Nature ſeemed to counteract it. The fiſtula broke out again, and the operation was again repeated, and the complaint complaint was ſoon apparently cured. But the patient was ſcarcely in a ſtate of convaleſcence, when the ſymptoms which had affected his breaſt again returned in ſuch an aggravated degree, that his ſiſter applied to me for advice. I employed leeches, veſicatories, and every other means preſcribed in ſimilar caſes, but in vain. Phthiſis pulmonalis made ſo rapid a progreſs, that this unfortunate gentleman died a few months after, of a conſumption.

### SEVENTH OBSERVATION.

An attorney of the Bailiwick of Réthel was during ſeveral days afflicted at different intervals, with ſpaſmodic colics, which often attacked him in the form of *cholera morbus*. He was ſuddenly troubled with an abſceſs, ſucceeded by a fiſtula, which only extended from 12 to 15 millimêtres (half an inch) in depth. I adviſed him not to attempt the cure of it precipitately, but to keep the part affected in the moſt eaſy ſtate, and to uſe ſimple injections. I kept the fiſtula open with prepared ſponge, and obſerved to the patient that the colics to which he was liable had not returned ſince his abſceſs happened. In this ſtate he lived for a year, without experiencing the leaſt change in his health; but he at length became impatient to be relieved from a ſtate more diſagreable than dangerous. The operation was performed by a ſkilful ſurgeon, who was not informed of the circumſtances which had preceded the fiſtula. Some time after his cure, the attorney obſerved a conſiderable change in his health; and the colics returned with ſuch violence, that in the month of January, 1787, an extreme paroxyſm cauſed his death.

Being called in to open the body, I found that all the viſcera of the abdomen had been affected by the inflammation; the inteſtines were in a gangrenous ſtate, they appeared to be entangled, and preſented an heterogenous maſs. No morbid appearance was perceptible in the kidneys, ureters, or bladder, which could juſtify the prevailing ſuppoſition that the patient's death had been occaſioned by long-continued nephritic attacks.

